# Occurrence of Typhoid Fever Complications and Their Relation to Duration of Illness Preceding Hospitalization: A Systematic Literature Review and Meta-analysis

**DOI:** 10.1093/cid/ciz477

**Published:** 2019-10-30

**Authors:** Ligia María Cruz Espinoza, Ellen McCreedy, Marianne Holm, Justin Im, Ondari D Mogeni, Prerana Parajulee, Ursula Panzner, Se Eun Park, Trevor Toy, Andrea Haselbeck, Hye Jin Seo, Hyon Jin Jeon, Jong-Hoon Kim, Soo Young Kwon, Jerome H Kim, Christopher M Parry, Florian Marks

**Affiliations:** 1 International Vaccine Institute, Seoul National University Research Park, Republic of Korea; 2 Center for Gerontology and Healthcare Research, School of Public Health, Brown University, Providence, Rhode Island; 3 Oxford University Clinical Research Unit, Ho Chi Minh City, Vietnam; 4 Department of Medicine, University of Cambridge, United Kingdom, United Kingdom; 5 Clinical Sciences, Liverpool School of Tropical Medicine, United Kingdom

**Keywords:** typhoid fever, complications, risk factors, meta-analysis, prevalence

## Abstract

**Background:**

Complications from typhoid fever disease have been estimated to occur in 10%–15% of hospitalized patients, with evidence of a higher risk in children and when delaying the implementation of effective antimicrobial treatment. We estimated the prevalence of complications in hospitalized patients with culture-confirmed typhoid fever and the effects of delaying the implementation of effective antimicrobial treatment and age on the prevalence and risk of complications.

**Methods:**

A systematic review and meta-analysis were performed using studies in the PubMed database. We rated risk of bias and conducted random-effects meta-analyses. Days of disease at hospitalization (DDA) was used as a surrogate for delaying the implementation of effective antimicrobial treatment. Analyses were stratified by DDA (DDA <10 versus ≥10 mean/median days of disease) and by age (children versus adults). Differences in risk were assessed using odds ratios (ORs) and 95% confidence intervals (CIs). Heterogeneity and publication bias were evaluated with the *I*^2^ value and funnel plot analysis, respectively.

**Results:**

The pooled prevalence of complications estimated among hospitalized typhoid fever patients was 27% (95% CI, 21%–32%; *I*^2^ = 90.9%, *P* < .0001). Patients with a DDA ≥ 10 days presented higher prevalence (36% [95% CI, 29%–43%]) and three times greater risk of severe disease (OR, 3.00 [95% CI, 2.14–4.17]; *P* < .0001) than patients arriving earlier (16% [95% CI, 13%– 18%]). Difference in prevalence and risk by age groups were not significant.

**Conclusions:**

This meta-analysis identified a higher overall prevalence of complications than previously reported and a strong association between duration of symptoms prior to hospitalization and risk of serious complications.

Typhoid fever is a disease caused by the bacterium *Salmonella enterica* serovar Typhi (*Salmonella* Typhi), which is transmitted through ingestion of food and water contaminated with feces from patients with typhoid fever or carriers [1]. Global estimates indicate that 11–20 million individuals are infected with the disease, with 120 000–220 000 dying annually [2]. The disease incidence is especially high in preschool children and infants [2–5]. Countries in South Asia have the highest incidence of the disease, though recent estimates suggest a comparatively substantial burden in African countries [2, 6, 7].

The progression of the illness without appropriate diagnosis and treatment may result in the development of complications, usually by the second to fourth week of illness [8]. Among patients hospitalized with typhoid fever, complications are estimated to occur in 10%–15% [1], with greater risk for complications among typhoid fever patients with one or more of the following risk factors: a delay in implementing an effective antimicrobial treatment [9], infection with an antimicrobial-resistant *Salmonella* Typhi strain [10], and very young age (infants) [11–16].

Despite the accumulating research on the development of complications associated with these risk factors, the increased risk and frequency of complications has not been systematically assessed. The objectives of the study were to estimate the overall prevalence of complications in hospitalized patients with culture-confirmed typhoid fever and to assess the effect of delaying the initiation of appropriate antimicrobial therapy and age on the prevalence and risk of complications.

## METHODS

### Search Strategy and Selection Criteria

We searched the PubMed database using the terms “typhoid fever,” “enteric fever,” “*Salmonella* Typhi,” “complication,” and “complications.” Results were restricted to publications in English, listed from 1 January 1990 to 31 December 2018, where abstracts were available for review and research was conducted in human subjects. Publications with confirmed typhoid fever cases by blood, bone marrow, and/or stool culture and with disaggregated data on typhoid fever complications were selected for full review. Letters, reviews, editorials, case reports, case series reporting only one type of complications or one system affected, and studies not relevant to the subject of research, as well as publications where complication for nontyphoidal *Salmonella* or paratyphoid could not be disaggregated from typhoid fever complications and studies reporting complications exported to developed countries, were excluded. The selection criteria and search terms for the study are listed in Table 1.

**Table 1.** Search Terms, Search Limits, and Selection Criteria for Study Inclusion From the PubMed Database Review

Search Terms
• ((typhoid fever) OR (enteric fever) OR (*Salmonella* Typhi)) AND (complications OR complication)
Search Limits
• Published in English in peer-reviewed journal from 1 January 1990 to 31 December 2018 • Abstracts available for review • Research conducted in human subjects
Selection Criteria
• Typhoid fever cases identified from prospective/retrospective, cross-sectional, or surveillance studies • Typhoid fever cases confirmed by blood culture, bone marrow culture, or stool culture • Listed complications identified from culture-confirmed typhoid fever cases

**Figure 1.**
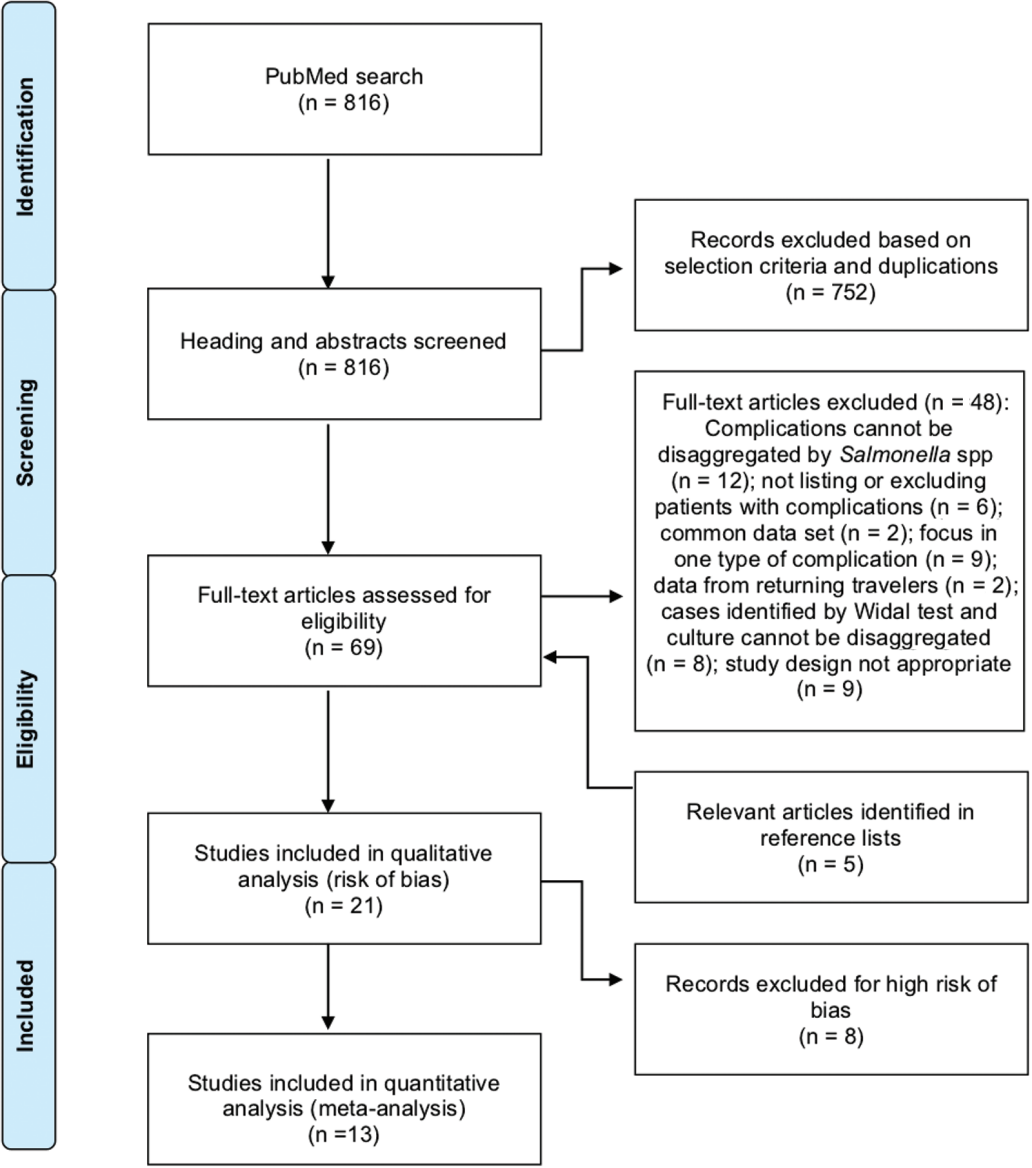
Study selection flow diagram (1990–2018).

Two independent researchers screened the title and abstract of resulting articles based on the criteria described above. Discrepancies were resolved by reviewing the full article and confirming the selection criteria. Articles considered as relevant for full review were downloaded and reviewed by one researcher. References were also screened to identify potential relevant articles not appearing in the initial search. Details of the search strategy are presented in Figure 1. No written protocol was published for this review.

### Risk of Bias of Individual Studies

A set of criteria was developed to assess the risk of bias (RoB) of each relevant article and ensure that methods were sufficiently similar across articles to perform the meta-analysis [17, 18]. The three types of bias assessed were selection bias, detection bias, and reporting bias. For selection bias, we assessed how the cases were identified (laboratory-confirmed versus clinical diagnosis); whether or not the authors established clear exclusion criteria for cases with confounding illnesses such as immunodeficiency, malignancy, major congenital abnormalities or syndromes, and chronic illnesses; and whether the exclusion criteria were based on prior report of antimicrobial therapy, presentation of complications at recruitment, or antimicrobial resistance (AMR) patterns. For detection bias, we assessed the potential for misclassification via unclear definitions of complications and inclusion of a reduced group of all cases identified for statistical analyses. For reporting bias, we assessed the completeness of reporting of complications of interest: whether all or a reduced group of complications observed were reported or not and whether key risk factors related to complications (days of disease before admission to the hospital and analysis of AMR) were reported. Based on these criteria, each article was classified as having low, medium, or high RoB (Table 2).

**Table 2.** Risk of Bias Assessment of Articles Selected for the Qualitative Analyses From the Systematic Literature Review (1990–2018)

RoB Assessment Criteria (All That Apply)	Study, First Author [Reference]																				
	Parry [19]	Malik [20]	Limpitikul [21]	Kadhiravan [22]	Abucejo [23]	Kabra [10]	Rajajee [12]	Khosla [24]	Walia [16]	Song [25]	Feasey [26]	Wongsawat [27]	Van den bergh [9]	Khan [28]	Rao [29]	Rasaily [30]	Ollé-Goig [31]	Seçmmer [32]	Sharma [33]	Mishra [34]	Mukherjee [35]
Selection^a^	L	L	M	M	M	M	M	M	H	M	M	H	L	L	M	H	L	M	H	M	M
Selection/recruitment of TF culture-confirmed cases																					
From overall hospital visits/lab records (L)	X	X	X	X	X	X	X	X	X			X	X	X	X		X	X		X	X
From patients with suspected typhoid fever /other criteria (M)										X	X					X					
Selection/ recruitment not described (H)																			X		
Exclusion of typhoid fever culture-confirmed cases																					
Exclusion criteria reported (L)	X	X											X	X			X				
Exclusion criteria not reported (M)			X	X	X	X	X	X		X	X				X			X	X	X	X
Based on AMR, pretreatment, or incomplete medical file (H)									X			X				X					
Identification and measurment of complications^a^	L	L	L	L	M	L	M	M	L	L	L	L	L	L	M	H	M	M	M	M	H
Definition of reported complications																					
All/some complications defined (L)	X	X	X	X		X			X	X	X	X	X	X		X					
No complications defined (M)					X		X	X							X		X	X	X	X	X
Complications measured from																					
All selected typhoid fever cases included for analysis (L)	X	X	X	X	X	X	X	X	X	X^b^	X	X	X	X	X		X	X	X	X	
Subset of selected typhoid fever cases included for analysis (H)																X					X
Reporting^a^	L	L	L	L	M	L	M	M	L	M	M	M	M	M	H	L	H	H	H	M	H
Complications reported																					
All observed complications counted and reported (L)	X	X	X	X		X	X	X	X	X	X	X	X			X				X	
All observed complications counted but not all reported (M)					X									X							
Some complications counted and reported (H)															X		X	X	X		X
Key risk factors/information to interpret result																					
AMR analysis and days of disease before admission/recruitment reported (L)	X	X	X	X	X	X			X							X			X		X
AMR analysis not reported (M)											X		X	X			X				
Days of disease before admission/recruitment not reported (M)							X	X		X	X	X	X		X			X		X	
Overall RoB^a^	L	L	M	M	M	M	M	M	H	M	M	H	M	M	H	H	H	H	H	M	H

Abbreviations: AMR, antimicrobial resistance; H, high; L, low; M, medium; RoB, risk of bias.

^a^For each subgroup (Selection, Detection, Reporting) and for the overall RoB assessment, RoB was decided based on the following criteria: All criteria evaluated with a “low” RoB, then RoB for that section is “low”; 1 criterion evaluated with a “medium” RoB, then RoB for that section is “medium”; 1 criteria evaluated with a “high” RoB, then RoB for that section is “high.” The results from each subgroup assessment were used for the overall RoB.

^b^Complications are not reported for 31% (39/127) of culture-confirmed typhoid fever cases identified during the study. However, the cases from which complications were reported were randomly selected (88/127) and for this reason, considered not biased and included in the meta-analysis.

### Data Extraction

Information on the overall occurrence of typhoid fever complications, days of disease at admission, antimicrobials used to assess resistance, and mortality reported in the articles included in the RoB analysis (n = 21) were extracted into an Excel spreadsheet (Microsoft, Redmond, Washington) and summarized in Table 3. Additionally, information on study objective, target population, recruitment, inclusion/exclusion criteria, definition of complications, and type of complications reported in each of these publications is presented in the Supplementary Material.

**Table 3.** Prevalence of Complications and Risk Factor Data From Hospitalized Typhoid Fever Cases by Study (1990–2018)

				Typhoid Fever Cases			Antimicrobial Susceptibility, No. (%)					
				Total	Complication	Mortality						
First Author, Year of Publication, Country [Reference]	Study Years	Age Group	Days of Disease at Admission	No.	No. (%)	No. (%)	Antimicrobial Tested^a^	AMR	MDR	NARS	FRS	Risk of Bias/Main Reason
Song, 2017, China [25]	2005–2014	All ages	NR	88	46/88 (52.3%)	0	Ampi, Chlor, Tetra, Nac, Genta, Cotri, Cefo, Cipro	47/88 (53.4%)	0	47/88 (53.4%)	46/88 (52%)	M/From patients with suspected typhoid fever/other criteria. Exclusion criteria NR; days ill prior to admission NR
Parry, 2014, Vietnam [19]	1993–1999	All ages	8 (6–11)^b^	581	90/581 (15.5%)	3/581 (0.5%)	Ampi, Cotri, Cipro, Ceft, Chlor, Oflox, Azit, Nac	506/581 (87.1%)	469/581 (80.7%)	NR	215/581 (37%)	L
Malik, 2002, Malaysia [20]	1993–1998	<14 y	11.6 (4–35)^c^	102	33/102 (32.4%)	0	Ampi, Cotri, Chlor	0	0	NT	NT	L
Limpitikul, 2014, Thailand [21]	2009–2011	<15 y	5 (4–7)^b^	368	49/368 (13.3%)	0	Amox, Ampi, Cotri, Cipro, Cefo, Ceft	0	0	NT	0	M/Exclusion criteria NR
Kadhiravan, 2005, India [22]	2001–2003	All ages	8 (4.8–14)^b^	60	11/60 (18.3%)	0	Amox, Cotri, Ceft, Cipro, Chlo, Nac	47/60 (78.3%)	22/60 (36.6%)	47/60 (78%)	0	M/Exclusion criteria NR
Abucejo, 2001, Philippines [23]	1994–1997	All ages	57% >1 wk	422	77/422 (18.2)	9/422 (2%)	Ampi, Cotri, Ceft, Chlor, Cipro, Oflox	0	0	NT	0	M/Exclusion criteria NR; No. of typhoid fever cases with complications registered but not all complications reported; no complications defined
Kabra, 2000, India [10]	NR	Children^d^	62% >1 wk	100	40/100 (40%)	0	Amox, Ampi, Cotri, Cipro, Cefo, Ceft, Chlor, Ceph, Genta, Fura	80/100 (80%)	80/100 (80%)	NT	0	M/Exclusion criteria NR
Rajajee, 1995, India [12]	1991–1992	<3 y	NR	71	33/71 (46%)	2/71 (2.8%)	Ampi, Amik, Chlor, Cotri, Ceftr, Cipro, Kana, Genta, Netro, Cefta, Cepha, Carb, Cefu, Cefo	43/71 (60.5%)	36/71 (51%)	NT	0	M/Exclusion criteria NR; no complications defined; days ill prior to admission not reported
Khosla, 1998, India [24]	1991–1992	≥15 y	NR	180	51/180 (28.3)	12/180 (6.7%)	Ampi, Amox, Cotri, Chlor, Cipro, Genta, Tetra	124/180 (68.6%)	18/180 (10%)	NT	5/180 (3%)	M/Exclusion criteria NR; no complications defined; days ill prior to admission NR
Walia, 2005, India [16]	2001–2003	All ages	77.2% >1wk	88	41/88 (46.6%)	4/88 (4.5%)	Ampi, Cotri, Chlor, Ceft, Cefi, Cipro, Nac	NR	26/88 (29.5%)	63/88 (71.5%)	NR	H/typhoid fever cases who had previously received quinolones or cephalosporin or macrolides or chloramphenicol were excluded
Feasey, 2015, Malawi [26]	2011–2013	All ages	NR	325	58/325 (17.8%)	7/325 (2%)	Ampi, Chlor, Cotri, Cefp, Cipro	NR	NR	NT	NR	M/ Exclusion criteria NR; days ill prior to admission and antimicrobial resistance analyses NR
Wongsawat, 2002, Thailand [27]	1986– 2000	<16 y	NR	14	6/14 (42.9%)	0	Ampi, Cotri, Chlor, Cefo, Ceft, Cipro, Imip	2/14 (14.2)	0	NT	0	H/26% of typhoid fever cases excluded for incomplete medical records
van den Bergh, 1999, Indonesia [9]	1989–1990	≥14 y	NR	105	14/105 (13.3%)	5/105 (5%)	NR	…	…	…	…	M/Days ill prior to admission and antimicrobial resistance analysis NR
Khan, 1999, South Africa [28]	1993–1995	All ages	NA^e^	102	39/102 (38%)	1/102 (1%)	NR	…	…	…	…	M/Antimicrobial resistance analysis NR
Seçmeer, 1995, Turkey [32]	1982–1992	<14 y	NR	27	NA^f^	4/27 (14.8%)	Ampi, Ceft, Cefo, Chlor, Cotri	3/27 (11.1%)	NR	NT	NT	H/Typhoid Fever cases with complications NR; only selected complications included in the article
Rasaily, 1994, India [30]	1990–1992	<12 y	16.4 (10)^g^	172	11/172 (6.4%)	3/172 (1.7%)	Ampi, Amik, Chlor, Cipro, Fura, Genta, Nac, Norf, Tetra, Cotri	172/172 (100%)	172/172 (100%)	4/172 (2.5%)	0	H/Typhoid Fever cases with complications at recruitment and/or infected with strains fully sensitive to antimicrobials were excluded
Ollé-Goig, 1993, Haiti [31]	1988–1991	≥14 y	95% <15 d	217	NA^f^	20/129 (9.25%)	NR	…	…	…	…	H/Typhoid Fever cases with complications NR; only selected complications included in the article
Rao, 1993, India [29]	1990–1991	NR	NR	102	NA^f^	1/102 (1%)	Ampi, Chlor, Tetra, Genta, Kana, Amox, Cotri, Cipro, Norf	80/102 (78.4%)	80/102 (78.4%)	NT	0	H/Typhoid Fever cases with complications NR; only selected complications included in the article
Sharma, 1993, India [33]	NR	<15 y	NA^e^	65	NA^f^	2/65 (3.1%)	Ampi, Chlor, Cotri, Genta, Norf	42/65 (64.6%)	42/65 (64.6%)	NT	0	H/Typhoid Fever cases with complications NR; only selected complications included in the article
Mishra, 1991, India [34]	1990	Children	NR	50	14/50 (28.2%)	1/50 (2%)	NR	…	39/50 (78%)	…	…	M/Exclusion criteria NR; complications not defined; days ill prior to admission not reported
Mukherjee, 1991, India [35]	1989–1990	All ages	76% >1wk	46	NA^f^	6/46 (13%)	Ampi, Chlor, Clox, Cotri, Cipro, Norf, Genta, Fura	32/46 (69.5%)	31/46 (67.3%)	NT	0	H/Typhoid Fever cases with complications NR; only selected complications included in the article

Abbreviations: AMR, *Salmonella* Typhi isolates resistant to at least 1 of the antimicrobials tested; FRS, isolates with reduced susceptibility or resistant to fluoroquinolones; H, high; L, low; M, medium; MDR, multidrug-resistant *Salmonella* Typhi isolates; NARS, nalidixic acid–resistant *Salmonella* Typhi; NA, not available; NR, not reported; NT, not tested.

^a^Azit (azithromycin), Amox (amoxicillin), Ampi (ampicillin), Amik (amikacin), Cotri (cotrimoxazole), Ceph (cephalexin), Cipro (ciprofloxacin), Cefo (cefotaxime), Ceft (ceftriaxone), Cefta (ceftazidime), Cefi (cefixime), Cefp (cefpodoxime), Chlor (chloramphenicol), Cepha (cephaloridine), Carb (carbenicillin), Cefu (cefuroxime), Cefo (cefotaxime), Clox (cloxacillin), Fura (furazolidone), Genta (gentamicin), Imip (imipenem), Kana (kanamicin), Nac (Nalidixic acid), Netro (netromycin), Norf (norfloxacin), Oflox (Ofloxacin), Tetra (tetracycline).

^b^Median (interquartile range).

^c^Mean (range).

^d^Upper age limit for children not described.

^e^Days of illness at admission reported stratified by a second variable (sex/MDR); it cannot be used to compare with days of illness at admission from others articles that report nonstratified days of illness.

^f^Complications are listed, but the publication does not specify the number of patients reporting these complications.

^g^Mean (standard deviation).

### Data Analysis

The main objective of this meta-analysis was to estimate a pooled prevalence of typhoid fever complications and, secondarily, to evaluate the effect of delaying appropriate antimicrobial therapy and age on the prevalence and risk of complications. The number of typhoid fever cases reported with complications and all culture-confirmed (blood, bone marrow, and/or stool culture) typhoid fever cases reported by each author were used in the meta-analysis. Any unfavorable evolution of the disease reported as a complication in a patient with typhoid fever in the publications included for analysis was considered a complication; as a consequence, the list of complications generated for this review is entirely driven by what each author reported. The different types of complications listed by each article are included in the Supplementary Material. Prevalence for each type of complication reported was estimated using as a numerator the sum of the frequencies for the specific complication and as the denominator the sum of all typhoid fever cases reported by the studies that measure the specific complication.

Description of individual-level data on antimicrobial susceptibility of the *Salmonella* Typhi isolates and timing from disease onset to initiation of therapy needed to evaluate whether a patient received appropriate antimicrobial therapy was not available in the literature reviewed. The average days from disease onset to hospitalization (DDA) reported by the studies included in the meta-analysis was used as a surrogate to assess the effect of delaying appropriate antimicrobial therapy on the prevalence and risk of complications.

**Figure 2.**
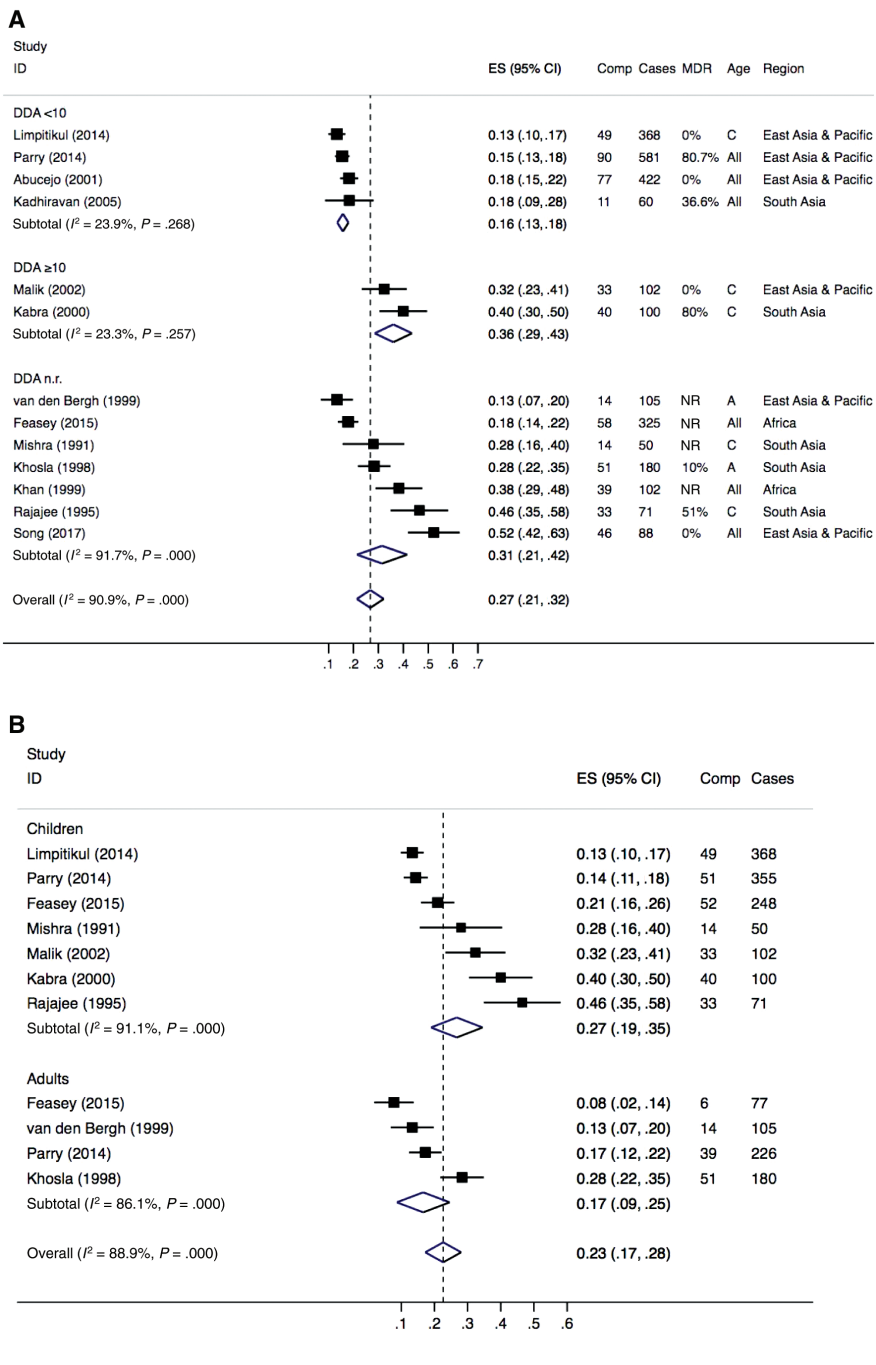
Forest plots showing the overall and subgroup prevalence of typhoid fever complications among hospitalized patients (1990–2018), by illness duration at hospitalization (*A*) and age (*B*). Abbreviations: A adults; All, all ages; C, children; CI, confidence interval; Comp, complications; DDA, mean/median illness duration (days) at hospitalization; ES, estimated prevalence; ID, study ID, study identification-first author (year of publication); MDR, multidrug antimicrobial resistance reported in each study; NR, not reported.

The literature describes that complications usually develop by the second to fourth week of illness among typhoid fever patients not receiving appropriate diagnosis and/or treatment [8]. However, a specific cutoff point to assess the effect of DDA on complications was not identified in the literature review. With the objective to identify a specific cutoff point, a preliminary analysis with the available information from six relevant (low or medium RoB studies reporting illness duration at hospitalization) articles was performed, focusing on the period from 2–4 weeks of illness duration. The results displayed differences in the prevalence of complications between studies with patients reporting an average of ≥10 days of disease onset at hospitalization and studies with patients reporting <10 days. The same result was observed after adding other articles with high RoB to the preliminary analysis. As a consequence, the 10-day cutoff point was applied to the subgroup analysis (Figure 2). The application of meta-regression to assess the effect of DDA on complications was not possible due to the few number of articles identified for this secondary analysis.

The mean or median days of disease at admission reported in each study were used to stratify data in two DDA groups. Group 1 included studies reporting a mean or median duration of disease of <10 days (DDA <10). Group 2 included studies reporting a mean or median duration of disease of ≥10 days (DDA ≥10). Studies not describing the mean or median days of disease at admission but reporting the proportion of cases arriving at the hospital within the first week of the disease were assigned to group 1 or 2 using the following criterion: Studies with ≥60% typhoid fever cases arriving during the second week or later of the disease were included in group 2; otherwise, studies were included in group 1.

The effect of age on the prevalence and risk of complications was assessed using the prevalence of complications reported for children and adults in the studies. Most studies included in the age group analysis reported complications in two age groups: children and adults. The age limits used to define the age group in each of these studies were used. Organizing the complications in newly defined age groups with a specific cutoff point between children and adults was not possible due to the different age limits used across studies; the upper age limit for children varied from 12 to 15 years, whereas the lower age limit for adults varied from 14 to 16 years.

Analyses were conducted in Stata software version 15.1 (StataCorp, College Station, Texas). Funnel plot analysis, with the Egger test, was used to assess for publication bias. We evaluated statistical significance at *P* <.05. Sensitivity analysis was done using studies with high RoB, not included in the meta-analysis. Additionally, odds ratios (ORs) and χ ^2^ tests were used to evaluate for differential risks of complications between the DDA and age groups.

The meta-analysis was performed using a random-effects model assuming there were differences between the studies that were not caused by random chance, for example, differences in populations and context in which the studies took place. The *I*^2^ statistic was used to measure the extent to which studies agreed with each other (heterogeneity). The estimated prevalence of typhoid fever complications and overlapping of their 95% confidence intervals (CIs) were visually inspected using forest plots, and heterogeneity between studies was verified in the overall and in the subgroup analysis. An *I*^2^ <30% was considered as representing low or unimportant heterogeneity; an *I*^2^ between 30% and 50% as moderate heterogeneity, and >50% as substantial/high heterogeneity [36].

## RESULTS

The initial search in PubMed retrieved 2,397 records. After limiting articles to those published in English, published in PubMed between 1 January 1990 and 31 December 2018, reporting studies conducted in humans, and where abstracts were available for review, 816 (34%) articles remained. Review of titles and abstracts narrowed down the list to 64 relevant studies (Figure 1). Sixteen studies met inclusion criteria after full text review, and five additional records were added by supplemental review of reference lists.

Twenty-one studies were assessed for RoB for the meta-analysis (Table 2). Five studies reported a list of typhoid fever complications observed but did not describe the total number of typhoid fever cases in which these complications were observed [29, 31–33, 35]. We did not assume that the total number of complications reported were equal to the total number of cases with complications as multiple complications can occur in a single patient. These studies were considered high RoB and were excluded from the meta-analysis. Three additional studies were excluded from the meta-analyses due to their selection criteria: one study excluded all typhoid fever cases found with complications at recruitment and/or infected with *Salmonella* Typhi strains fully susceptible to the antimicrobials tested by the authors [30]; the second study excluded patients with typhoid fever who received quinolones or cephalosporin or macrolides or chloramphenicol prior to enrollment [16]; and the third study excluded 26% of typhoid fever culture-confirmed cases due to incomplete medical files, reducing the total number of typhoid fever cases included in the analysis [27]. We believed that these study characteristics created a unique and different group of typhoid fever cases that could not be compared with the rest of the studies, which included typhoid fever cases independent of resistance, development of complications, or pretreatment. These studies were, therefore, considered to have high RoB and were excluded from the meta-analysis. Finally, 13 studies, with low or medium RoB, were included in the meta-analysis [9, 10, 12, 19–26, 28, 34]. Information extracted from the 21 publications included in the RoB analysis is presented in Table 3 and Supplementary Material.

The 13 studies selected for the meta-analysis originated from seven countries in Asia (Thailand, Vietnam, Malaysia, Philippines, India, Indonesia, and China) and two countries in Africa (Malawi and South Africa). The studies included a total of 2,554 patients with typhoid fever, with 555 incurring complications. Five studies included only children [10, 12, 20, 21, 34], two included only adults [9, 24], and six included individuals from all ages [19, 22, 23, 25, 26, 28], with two of them reporting complication by age groups [19, 26] (Table 3). Thirteen studies were used to calculate the overall pooled prevalence of typhoid fever complications, nine to perform subgroup analysis by age group (seven for children, four for adults), and six to perform subgroup analyses by DDA groups (four studies with DDA <10, two studies with DDA ≥10). Four of the seven studies reporting complications in children provided information for duration of illness at hospitalization. These four studies were used to stratify the analysis by duration of illness (DDA groups) and explore differences in risk. Due to the limited number of publications, no analysis could be performed by AMR.

Further stratified analysis of DDA by region was not performed due to the small number of studies that provided this information (n = 6). Four of the six studies included in the subgroup analysis by DDA originated from East Asia and Asia Pacific and two from South Asia (Figure 2A); none of the studies from Africa provided information on symptom duration. Within Asia, the two studies from South Asia reported the highest prevalence of complications for both DDA groups.

### Overall Prevalence of Typhoid Fever Complications

Pooled analysis from the 13 studies resulted in an estimated 27% (95% CI, 21%–32%) of all blood culture–confirmed typhoid fever cases resulting in complications. A very high heterogeneity (*I*^2^ = 90.9%, *P* = .000) was found, indicating that the occurrence of typhoid fever complications reported was not consistent across the different studies (Figure 2A).

The heterogeneity was reduced when studies were grouped by DDA, presenting fairly consistent estimates of the occurrence of typhoid fever complications within each group, despite variations in multidrug resistance and age (Figure 2A). The pooled prevalence of typhoid fever complications in studies reporting DDA ≥10 was higher (36% [95% CI, 29%–43%]) than the pooled prevalence from studies reporting DDA < 10 (16% [95% CI, 13%–18%]). Additionally, the odds of developing complications were three times greater in studies reporting DDA ≥10 compared with those seeking care earlier (OR, 3.00 [95% CI, 2.14–4.17]; *P* < .0001).

The analysis by age group showed a higher pooled prevalence of typhoid fever complications from studies including children (27% [95% CI, 19%–35%]) than from studies including adults (17% [95% CI, 9%–25%]), with a very high heterogeneity for both groups (Figure 2B). The increased odds of developing complications observed in children was not significant (OR, 1.15 [95% CI, .89–1.49]; *P* = .247). A subgroup analysis by DDA in the group of studies with children showed a higher prevalence of complications in children reporting DDA ≥10 compared to those seeking care earlier (36% [95% CI, 29%–43%] vs 14% [95% CI, 11%–16%]), translating in three times greater odds of developing complications for children with prolonged disease at hospitalization than children with shorter disease duration (OR, 3.25 [95% CI, 2.42–5.10]; *P* < .000). A stratified analysis by DDA in the group of studies with adults was not possible as only two of the four studies reported a DDA <10 and the other two studies did not provide information on illness duration at hospitalization.

The Egger test for the prevalence analysis was not suggestive of the presence of publication bias (*P* = .259 for Figure 2A and *P* = .287 for Figure 2B).The sensitivity analyses conducted with two additional high RoB publications [16, 27], one of them with information on days of disease before hospitalization [16], resulted in similar pooled prevalences of typhoid fever complications (overall prevalence, 29% [95% CI, 23%–34%]; DDA <10, 16% [95% CI, 13%–18%]; DDA ≥10, 39% [95% CI, 31%–47%]).

### Occurrence of Specific Complications

**Figure 3.**
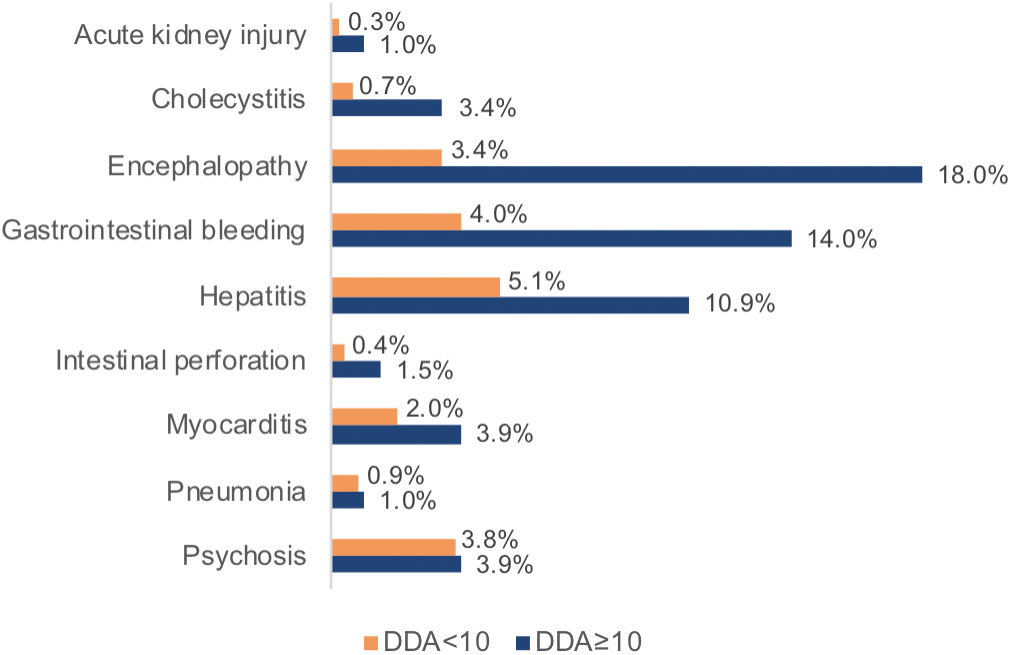
Frequency (pooled across the studies reporting the complications and illness duration at hospitalization) of typhoid fever complications (1990–2018). Abbreviation: DDA, mean/median illness duration at hospitalization.

The overall prevalence of each type of typhoid fever complication reported by the studies included in the meta-analysis, estimated using a random-effects model, is presented in Table 4. Overall, the complications with the highest prevalence (95% CI) reported by at least three studies are encephalopathy, gastrointestinal bleeding, and nephritis, with a prevalence of 7.3% (2.8%–11.9%), 5.7% (2.4%–9.0%), and 4.8% (0.1%–9.4%), respectively. When stratified by DDA group, the prevalence of complications reported in both groups differed (Figure 3). Hepatitis (5.1%) and gastrointestinal bleeding (4.0%) were the two most frequent complications reported in studies included in the DDA<10 group, whereas encephalopathy (18%) and gastrointestinal bleeding (14%) were the two most frequent complications in the studies from the DDA ≥10 group. Results from comparing the prevalence of complications reported in both groups (Table 5) revealed a higher risk for complications with DDA ≥10, with significant results for encephalopathy (OR, 6.81 [95% CI, 3.24–14.13]), cholecystitis (OR, 5.17 [95% CI, 1.29–24.32]), gastrointestinal bleeding (OR, 2.98 [95% CI, 1.46–5.70]), and hepatitis (OR, 2.36 [95% CI, 1.29–4.27]).

**Table 4.** Frequency and Prevalence^^a^^ of Specific Typhoid Fever Complications Reported From Articles Included in the Meta-analysis (1990–2018)

Complication	Frequency, No.	Total Group of Typhoid Fever Cases From Which Complications Are Reported, No.	Prevalence^b^, % (95% CI)	Study Reporting the Complication, First Author [Reference]
Aminotransferase elevation	28	88	32 (22–42)	Song [25]
Acute kidney injury	2	470	0.3 (–.2 to .8)	Malik [20], Limpitikul [21]
Anemia	41	693	5.8 (4–7.8)	Limpitikul [21], Feasey [26]
Ascites	7	368	1.9 (.5–3.3)	Limpitikul [21]
Blood transfusion	3	581	0.5 (–.1 to 1.1)	Parry [19]
Bone marrow suppression	8	102	7.8 (2.6–13.1)	Malik [20]
Bleeding or perforation	3	105	2.9 (–.3 to 6)	van den Bergh [9]
Confusion	5	325	1.5 (.2–2.9)	Feasey [26]
Changes in sensorium	3	60	5.0 (–.5 to 10.5)	Kadhiravan [22]
Cholecystitis	18	854	3.2 (.2–6.1)	Malik [20], Kabra [10], Rajajee [12], Parry [19]
Cyanotic episodes	3	71	4.2 (–.5 to 8.9)	Rajajee [12]
Decreased consciousness level	13	325	4.0 (1.9–6.1)	Feasey [26]
Encephalopathy	47	879	7.3 (2.8–11.9)	Kabra [10], Parry [19], Kadhiravan [22], Mishra [34] Song [25]
Gastrointestinal bleeding	75	1251	5.7 (2.4–9.0)	Kabra [10], Parry [19], Kadhiravan [22], Abucejo [23], Song [25]
Hemorrhage	4	180	2.2 (.1–4.4)	Khosla [24]
Hemolysis	1	102	1.0 (–.9 to 2.9)	Malik [20]
Hemodynamic shock	5	581	0.9 (.1–1.6)	Parry [19]
Hemolytic anemia	3	368	0.8 (–.1 to 1.7)	Limpitikul [21]
Hepatitis	71	1500	4.9 (2.3–7.5)	Malik [20], Kabra [10], Parry [19], Khosla [24], Kadhiravan [22], Feasey [26], Mishra [34], Khan [28]
Hypothermia	10	71	14.1 (6.0–22.2)	Rajajee [12]
Ileitis	21	422	5.0 (2.9–7.1)	Abucejo [23]
Intestinal perforation	32	2268	1.1 (.4–1.8)	Malik [20], Limpitikul [21], Kabra [10], Parry [19], Khosla [24], Abucejo [23], Feasey [26], Khan [28], Song [25]
Lower respiratory symptoms	7	368	1.9 (.5–3.3)	Limpitikul [21]
Liver abscess	1	71	1.4 (–1.3 to 4.1)	Rajajee [12]
Marrow hypoplasia	6	71	8.5 (2.0–14.9)	Rajajee [12]
Meningitis	4	131	2.5 (–.2 to 5.2)	Rajajee [12], Kadhiravan [22]
Myocarditis	41	1491	2.8 (1.5–4.0)	Malik [20], Parry [19], Khosla [24], Kadhiravan [22], van den Bergh [9], Feasey [26], Mishra [34], Song [25]
Neuropsychiatric complications	24	180	13.3 (8.4–18.3)	Khosla [24]
Nephritis/glomerulonephritis	16	273	4.8 (.1–9.4)	Kabra [10], Rajajee [12], Khan [28]
Osteomyelitis	2	102	2.0 (–.7 to 4.7)	Malik [20]
Paralytic ileus	12	282	4.2 (.4–8.1)	Malik [20], Khosla [24]
Peripheral circulatory failure	2	180	1.1 (–.4 to 2.6)	Khosla [24]
Peritonitis	2	88	2.3 (–.8 to 5.4)	Song [25]
Pleural effusion	7	631	1.1 (.3–1.9)	Parry [19], Mishra [34]
Pneumonia/bronchopneumonia	32	1234	2.5 (.7–4.2)	Malik [20], Parry [19], Rajajee [12], van den Bergh [9], Feasey [26], Mishra [34]
Psychosis	20	524	3.8 (2.2–5.5)	Malik [20], Abucejo [23]
Renal impairment	4	581	0.7 (.0–1.4)	Parry [19]
SIADH	2	102	2.0 (–.7 to 4.7)	Malik [20]
Seizure	1	368	0.3 (–.3 to .8)	Limpitikul [21]
Sepsis syndrome	10	105	9.5 (3.9–15.1)	van den Bergh [9]
Severe anemia	6	581	1.0 (.2–1.9)	Parry [19]
Stupor or coma	3	105	2.9 (–.3 to 6.0)	van den Bergh [9]
Transient thrombocytopenia	4	100	4.0 (.2–7.8)	Kabra [10]

Abbreviations: CI, confidence interval; SIADH, syndrome of inappropriate antidiuretic hormone.

^a^Includes data from publications that measured the specific complication.

^b^Prevalence estimated using a random-effects model.

**Table 5.** Risk Analyses^^a^^ of Typhoid Fever Complications Among Hospitalized Patients Reporting Illness Duration at Hospitalization (1990–2018)

	DDA ≥10				DDA <10					
Complication	Frequency, No.	Typhoid fever Cases, No.	Prevalence^b^, %	[Reference]	Frequency, No.	Typhoid fever Cases, No.	Prevalence^b^, %	[Reference]	OR (95% CI)	*P*-Value
Acute kidney injury	1	102	1.0	[20]	1	368	0.3	[21]	3.63 (.04–285.97)	.330
Cholecystitis	7	202	3.4	[10, 20]	4	581	0.7	[19]	5.17 (1.29–24.32)	.003
Encephalopathy	18	100	18.0	[10]	20	641	3.4	[19, 22, 23]	6.81 (3.24–14.13)	.000
GI bleeding	14	100	14.0	[10]	55	1063	4.0	[19, 22, 23]	2.98 (1.46–5.70)	.000
Hepatitis	23	202	10.9	[10, 20]	33	641	5.1	[19, 22]	2.36 (1.29–4.27)	.001
Intestinal perforation	3	202	1.5	[10, 20]	8	1371	0.4	[19, 21, 23]	2.56 (.43–10.80)	.151
Myocarditis	4	102	3.9	[20]	13	641	2.0	[19, 22]	1.97 (.45–6.54)	.234
Pneumonia	1	102	1.0	[20]	5	581	0.9	[19]	1.14 (.02–10.34)	.904
Psychosis	4	102	3.9	[20]	16	422	3.8	[23]	1.03 (.24–3.30)	.950

Abbreviations: CI, confidence interval; DDA, mean/median illness duration at hospitalization; GI, gastrointestinal; OR, odds ratio.

^a^Includes data from publications that measured the specific complication.

^b^Prevalence estimated using a random-effects model.

## DISCUSSION

### Occurrence of Typhoid Fever Complications

Few publications met the criteria required for inclusion in this meta-analysis, highlighting the limited high-quality evidence available on typhoid fever complications in the published literature. Overall, the pooled results showed a higher prevalence (27% [95% CI, 21%–32%]) of typhoid fever complications among hospitalized patients than previously reported (10%–15%) [1]. The stratified analyses revealed a higher prevalence of complications among patients reporting a mean/median of ≥10 days of disease (36% versus 16%), with three times the risk for developing complications compared to patients reporting a mean/median of <10 days of disease onset at hospital admission. This higher prevalence and risk was also observed among specific complications such as hepatitis, gastrointestinal bleeding, encephalopathy, and cholecystitis.

Consistent with the literature reporting young age as a risk factor for disease severity [11–16], the analysis by age group showed a higher prevalence of complications from studies including children than from studies including adults (27% versus 17%, respectively). Nevertheless, the increased risk observed in children was not statistically significant. The stratified analysis by illness duration (DDA groups) showed a three times higher odds for developing complications among children with a DDA ≥10 than among children with a DDA <10. This higher risk identified in children is similar to the risk identified in the analysis by DDA groups performed without taking into account age groups. These results suggest that, among children, there is also an increased risk of developing complications with a prolonged disease duration before hospitalization. However, it is important to highlight that across all studies included in the analysis, with and without data on illness duration, the highest prevalence of complications are reported in children, especially infants. We consider that incorrect diagnosis prior to hospitalization or delaying seeking care may lead to this disparity in complications at presentation to the hospital, as might the use of empirical antimicrobial agents to which the infecting *Salmonella* Typhi strain is resistant.

Assessment of the impact of AMR on the prevalence of typhoid fever complications was not performed in this study. In the literature, however, patients infected with an antimicrobial-resistant *Salmonella* Typhi strain experience more complications and greater disease severity in most studies [10, 12, 16, 22, 24, 33, 35]. A common factor that stands out in these studies, when reported, is the long duration of illness (mean/median of ≥10 days) before hospitalization [10, 16, 22, 33, 35]. This common factor is consistent with the results of the meta-analysis, leading us to postulate that AMR could increase the occurrence of complications by allowing the disease to progress at least to ≥10 days before proper diagnosis and effective treatment are implemented.

Whether the increased prevalence of complications observed among typhoid fever patients with ≥10 days of disease is due to the use of empirical antimicrobial agents to which the infecting organism is resistant needs further research. Overall, antimicrobial use prior to hospitalization was poorly documented among the articles reviewed and could not be evaluated here. Future research should disentangle the effects of delayed diagnosis from incorrect/ineffective first-line treatment and the effect of age on the development of complications. This would require consistent reporting of empirical antimicrobial use prior to hospitalization, days of disease at hospital admission, antimicrobial-resistant patterns, and presentation of complications in >2 age-stratified groups, information not reported in most studies at present.

### Mortality

Although not the focus of this meta-analysis, mortality was reported in eight of the 13 included studies. The mortality among the typhoid fever patients was low despite the high occurrence of complications described in some studies. Among the publications reporting deaths, fatal outcomes occurred among patients of all ages, with a mortality ranging from 0.5% (3/581) [19] to 6.7% (12/180) [24]. This mortality is similar to the median (range) case fatality rate reported from hospital-based studies by Crump et al in 2007 (2.0% [0–14.8%]) [4]. It is important to highlight that all patients included in this meta-analysis were followed up and received appropriate treatment in a hospital setting, two critical factors that likely contributed to the low mortality observed among the complicated typhoid fever cases.

### Limitations

The few number of articles included in the meta-analysis for the estimation of the overall and subgroup prevalence of complications is one of the main limitations to the results of this study. Only Thirteen of the relevant articles (Table 2) identified with the systematic review were included in the meta-analysis due to differences in the methods to select typhoid fever cases, measure complications, and report complications. The low number of studies included in the analysis also limits the investigation of reporting bias.

In addition to the scarcity of quality data, not all studies consistently report prior empirical antimicrobial use; days of disease at hospital admission; AMR patterns; the definitions used to measure complications, which is a critical factor that influences the prevalence of complications reported; and the occurrence of complications stratified by age group.

Additionally, the capacity to diagnose some complications, such as availability of ultrasound, electrocardiography, and laboratory tests to diagnose cholecystitis, myocarditis, hepatitis, and so on, may vary substantially between sites and influence reported complication rates. From the 21 relevant articles, only one study described the criteria used to measure and identify each of the complications observed and reported [19]; twelve studies reported days of disease or fever at hospitalization [10, 16, 19–23, 28, 30, 31, 33, 35]; four studies lacked the report of antimicrobial analysis [9, 26, 28, 31]; three studies reported prior antimicrobial use [20, 22, 23]; three of the seven studies including cases from all ages reported the occurrence of complications categorized by at least two age groups [16, 19, 26]; and five reported complications but not the number of patients with complications [29, 31–33, 35]. These data gaps limited the assessment of the impact of risk factors on complications.

We further highlight the limited publications on typhoid fever complications from Africa and South America and the variability of antimicrobials used to assess resistance. While multidrug resistance was reported in 15 of the 21 articles reviewed, susceptibility to nalidixic acid was assessed in only five studies [16, 19, 22, 25, 30]. Resistance to nalidixic acid or intermediate resistance to fluoroquinolones, rather than multidrug resistance, has been associated with typhoid fever severity and complications [16, 19]. Future studies should test for decreased susceptibility, or intermediate resistance, to fluoroquinolones in Salmonella Typhi, by testing for nalidixic acid or pefloxacin resistance or by ciprofloxacin minimum inhibitory concentrations, and report complications stratified by antimicrobial susceptibility pattern of infecting isolates. Furthermore, the pathogenic potential of the strain genotype could also affect the severity of typhoid fever [19]; however, that information is not routinely collected or reported.

Another limitation is the setting in which these studies took place. All studies were hospital-based and, as such, the criteria for hospital admission is likely to vary across studies and there is an increased risk of overestimating the occurrence of complications by measuring complications in typhoid fever patients with severe disease and access to healthcare [37]. Furthermore, limiting the report of complications to patients with culture-positive cases may result in bias toward more severe cases. Differences in geographical regions could not be assessed due to the limited number of published articles identified with enough quality to be included in the meta-analysis.

Last, the subgroup analysis by DDA and age group is based on aggregated rather than individualized data, which enabled comparisons across studies but not within studies. As a surrogate for delaying the implementation of effective antimicrobial treatment, we utilized mean/median DDA to dichotomize studies for comparison, using a threshold of 10 days; however, it is possible that most complications occur among individuals with much longer DDA and that our comparison underestimated the effects of disease duration on complications. A meta-regression could not be performed due to the few number of eligible articles (with low or medium RoB and reporting DDA data) included for this analysis (n = 6). For the age group analysis, we used the groups (children and/or adults) reported by the studies and not a standard cutoff point due to limited age-stratified information for complications that would allow us to organize cases in defined age groups. This approach may have underestimated the effect of age on the prevalence and risk of complications, especially in very young children. Lack of data on duration of illness also limited the stratification of adults by this variable.

## CONCLUSIONS

This systematic literature review found limited quality evidence concerning culture positive typhoid fever complications in the published literature. The meta-analysis identified a higher overall prevalence of complications than previously reported with a higher risk of typhoid fever complications for patients, including children, with a prolonged duration of disease prior to hospitalization, possibly driven by a delay in hospitalization and the administration of effective antimicrobial treatment. Typhoid fever cases from studies reporting a mean/median of ≥10 days of symptom duration at hospitalization not only have a higher prevalence of complications (36% versus 16%), but also three times the risk for developing complications compared with typhoid fever patients from studies reporting a mean/median of <10 days of symptoms at hospitalization. This higher risk was also observed from studies including children and reporting duration of illness at hospitalization. However, the results of this study should be interpreted with caution because of the limited number of publications included, as many articles were excluded for high RoB.

These results underscore the importance of adequate health infrastructure with early access to diagnosis and adequate treatment among patients with typhoid fever to reduce complications and severity of the disease. In high-risk countries with high AMR prevalence and limited access to healthcare, prevention and control strategies should be comprehensive and include reduction of barriers to healthcare access, improved diagnostics, and increased availability of new vaccines recommended by the World Health Organization [38].

## Supplementary Data

Supplementary materials are available at *Clinical Infectious Diseases* online. Consisting of data provided by the authors to benefit the reader, the posted materials are not copyedited and are the sole responsibility of the authors, so questions or comments should be addressed to the corresponding author.

ciz477_suppl_Annex_1_articles_included_in_RoB_analysis

ciz477_suppl_Annex_2_PRISMA_Checklist

## Notes

***Acknowledgments.*** The authors are grateful to Ji Hyun Han of the International Vaccine Institute (IVI) for programmatic support.

***Financial support.*** This research was funded by the Bill & Melinda Gates Foundation [OPP1127988]. The International Vaccine Institute acknowledges its donors including the Republic of Korea and the Swedish International Development Cooperation Agency. This publication was made possible through a grant from the Bill & Melinda Gates Foundation [OPP1201031].

***Supplement sponsorship.*** This article was published as part of the supplement “Severe Typhoid Fever in Africa (SETA) Program” sponsored by the International Vaccine Institute.

***Potential conflicts of interest.*** All authors report no potential conflicts of interest. All authors have submitted the ICMJE Form for Disclosure of Potential Conflicts of Interest. Conflicts that the editors consider relevant to the content of the manuscript have been disclosed.
