# Supplementary material for: Occurrence of Typhoid Fever Complications and Their Relation to Duration of Illness Preceding Hospitalization: A Systematic Literature Review and Meta-analysis
Source: Clin Infect Dis. 2019 Oct 30;69(Suppl 6):S435–48. doi: 10.1093/cid/ciz477 (PMC6821330; doi:10.1093/cid/ciz477)
Supplement: ciz477_suppl_Annex_1_articles_included_in_RoB_analysis [file ciz477_suppl_annex_1_articles_included_in_rob_analysis.docx]

**Annex 1 Additional information of articles included in the qualitative analysis**

| **STUDY INFO** | **METHODS** |  | **RESULTS** |  |
| --- | --- | --- | --- | --- |
| **Author, year [ref#]**  **Country**  **Study period (months)**  **Target population**  **Healthcare facility**  **Data collection** | **Objective**  **Recruitment/Selection**  **Inclusion criteria**  **Exclusion criteria**  **Definition of complications**  **Reporting of complications**  **Antimicrobials included for susceptibility analysis** | **Typhoid fever cases, N**  **Sex-male, Sn (%)**  **Age**  **Days of disease/fever, DDA/DFA**  **Pre-treatment, n (%)** | **Complications^1^ (Cn), n (%)**  **>1 Complication^2^ (MCn), n (%)**  **Mortality (Mn), n (%)**  **AMR^3^, n (%)**  **MDR^4^, n (%)**  **NARS^5^, n (%)**  **RFS^6^, n (%)** | **Complication listed by authors (n)** |
| **Song, 2017**  China  2005-2014 (120)  All ages  Hospital  Prospective | - To investigate the etiological characteristics of *Salmonella* Typhi isolates, including the genetic distribution by means of pulsed-field gel electrophoresis (PFGE) and resistance development, and to assess the clinical efficacy of antimicrobial treatment and complications in culture-confirmed patients. - Cases reported by the infectious disease surveillance system in Ningbo during 2005-2014. - Hospitalized patients with a sustained fever along with characteristic features, such as malaise, lethargy, myalgia, and decreased white blood cells, and who were clinically diagnosed as having typhoid fever and subsequently confirmed by culture or a fourfould increase in antibody titer by the Widal test. - Exclusion criteria not reported. - Some complications defined. - Complications reported and listed from 88/127 randomly selected culture confirmed typhoid fever patients. - Ampicillin, chloramphenicol, tetracycline, nalidix acid, gentamicine, trimethoprim- sulphamethoxazole, cefotanine, ciprofloxacin. | N=88  Sn= 50 (56.8%)  Age-Mean (range)= 34.3 (9-77y)  DDA=n.r.  Pre-treatment=n.r. | Cn= 46 (52.3%)  MCn=n.r.  Mn=0  AMR=47 (53.4%)  MDR=0  NARS=47 (53.4%)  RFS=46 (52.3%) | Aminotransferasa elevation (28)  Toxic myocarditis (6)  Encephalopathy (3)  Peritonitis (2)  Intestinal hemorrhage (6)  Intestinal perforation (1) |
| **Feasey, 2015**  Malawi  2011-2013 (25)  All ages  Hospital  Retrospective | - To report the emergence of a rapid and sustained increase in MDR-typhoid fever. - Review of medical files of a subset of typhoid fever patients recruited during a febrile surveillance project. - Patients with blood culture confirmed typhoid fever. - Exclusion criteria not reported. - Some complications defined. - Complications reported and listed from the typhoid fever patients selected for restrospective analysis of clinical files. - Ampicillin, chloramphenicol, cotrimoxazole, cefpodoxime, ciprofloxacin. | **Children < 16 years**  N=248  **Adults ≥ 16 years**  N=77  **All ages**  Sn=n.r.  Age-Mean/Median=n.r.  DDA=n.r.  Pre-treatment=n.r. | **Children < 16 years**  Cn= 52 (21%)  Mn=7 (2.8%)  **Adults ≥ 16 years**  Cn=6 (7.8%)  Mn= 0  **All ages**  MCn=n.r.  AMR=n.r.  MDR=n.r.  NARS=n.t.  RFS=n.r. | **Children < 16 years**  Anaemia requiring transfussion (16)  Meningism or decreased conscious level (13)  Pneumonia (12)  Intestinal perforation/peritonitis (9)  Hepatitis (1)  Myocarditis (4)  **Adults ≥ 16 years**  Confusion (5)  Intestinal perforation (1) |
| **Limpitikul, 2014**  Thailand  2009-2011 (22)  Children  Hospital  Retrospective | -To determine the clinical manifestations and outcomes, the reliability of *Salmonella* Typhi IgM and IgG rapid tests, and the susceptibility patterns and the response to treatment during the 2009-2011 typhoid outbreak in Songkhla province in Thailand.  -Reviewed of medical files of all children from 3 hospitals during the study period.  -Children with blood culture confirmed typhoid fever.  -Exclusion criteria not reported.  -Some complications defined.  -Complications reported and listed from all included typhoid fever cases.  -Amoxicillin, ampicillin, cefotaxime, ceftriaxone, co-trimoxazole, ciprofloxacin. | N=368  Sn= 165 (44.8%)  Age–Mean (SD), 7.5 (3)  DDF–Median (IQR), 5 (4-7)  Pre-treatment=n.r. | Cn= 49 (13.3%)  MCn= 5 (1.4%)  Mn= 0  AMR= 0  MDR= 0  NARS= n.t.  RFS= 0 | Ascites with abdominal pain (7)  Ileal perforation (1)  Wheezing (7)  Anemia (25)  Hemolytic anemia (3)  Thrombocytopenia (15)  Acute kidney injury (1) |
| **Parry, 2014**  Vietnam  1993-1999 (50)  All ages  Hospital  Prospective | - To determine microbiological and clinical factors associated with increased risk of severe typhoid fever, hypothesising that the changing antimicrobial resistance phenotypes throughout the selected sutdy periods in Vietnam were associated with shift in disease severity.  - Consecutive patients with blood or bone marrow culture confirmed typhoid fever were included in the study.  - Patients with blood or bone marrow culture confirmed typhoid fever.  - Excluded: patients with paratyphoid.  - All complications defined.  - Complications reported and listed from all included typhoid fever cases.  - Chloramphenicol, ampicillin, trimethoprim- sulphamethoxazole, ceftriaxone, ofloxacin, azithromycin, nalidixic acid, ciprofloxacin. | **Children < 16 years**  N=355  Sn= 196 (55.2%)  Age <16 y  DDA–Median (IQR), 7 (6-10)  Pre-treatment=n.r.  **Adults ≥ 16 years**  N=226  Sn= 100 (44.2%)  Age ≥ 16 y  DD–Median (IQR), 9 (7-14)  Pre-treatment=n.r. | **Children < 16 years**  Cn= 51 (14.3%)  MCn=n.r.  Mn= 0  AMR= 321 (90.4%)  MDR= 300 (84.5%)  NARS=n.r.  RFS= 144 (40.5%)  **Adults ≥ 16 years**  Cn= 39 (17.3%)  MCn=n.r.  Mn= 3 (1.3%)  AMR= 41 (81.8 %)  MDR= 169 (74.7%)  NARS= n.r.  RFS= 71 (31.4%) | **Children < 16 years**  Gastrointestinal bleeding (23)  Intestinal perforation (2)  Encephalopathy (8)  Myocarditis (9)  Hepatitis (19)  Cholecystitis (3)  Pneumonia (3)  Pleural effusion (4)  Severe anaemia (5)  Blood transfussion (2)  **Adults ≥ 16 years**  Bleeding (20)  Intestinal perforation (4)  Encephalopathy (8)  Myocarditis (3)  Hepatitis (10)  Cholecystitis (1)  Pneumonia (2)  Pleural effusion (2)  Haemodynamic shock (5)  Severe anaemia (1)  Blood transfussion (1)  Renal impairment (4) |
| **Wongsawat, 2002**  Thailand  1986-2000 (180)  Children  Hospital  Retrospective | - To determine the natural history of typhoid fever in pediatric patients during the last 15 years period at King Chulalongkorn Memorial Hospital. - Review of medical records of typhoid fever cases. - Patients with culture confirmed typhoid fever. - Excluded: typhoid fever patients with incomplete medical records (5/19, 26%). - Some complications defined. - Complications reported and listed from all included typhoid fever cases. - Co-trimoxazole, ampicillin, chloramphenicol, cefotaxime, ceftriaxone, ciprofloxacin, imipenem. | N=14  Sn= 9 (64.3%)  Age–Mean (range), 8.8 (2–15)  DDA=n.r.  Pre-treatment=n.r. | Cn= 6 (42.9%)  MCn=n.r.  Mn= 0  AMR=2 (14.2%)  MDR=0  NARS=n.t.  RFS=0 | Abnormal urine sediments (2)  Nephritis (1)  Severe enteritis (2)  Acute hemolysis (1) |
| **Malik, 2002**  Malaysia  1993-1998 (59)  Children  Hospital  Prospective | - To study the incidence and nature of complications of typhoid fever in children and also to identify the risk predictors/markers for these complications.  -Children with bacteriologically confirmed typhoid fever admitted to the hospital during the study period were recruited.  -Children with culture confirmed typhoid fever  -Excluded: typhoid fever cases with immune deficiency, malignancy, major congenital abnormalities or syndromes, chronic illnesses such as TB or chronic renal failure or those receiving steroids.  - Some complications defined.  -Complications reported from all cases.  -Ampicillin, Chloramphenicol, Co-trimoxazole. | N=102  Sn= 56 (54.9%)  Age–Mean (range),  91.3 months (6-159)  DDA–Mean(range),  11.5 (1-35)  Pre-treatment=51 (50%) | Cn= 33 (32.4%)  MCn=12 (11.7%)  Mn= 0  AMR= 0  MDR= 0  NARS= n.t  RFS= n.t. | Psychosis (4)  Myocarditis (4)  Pneumonia (1)  Paralytic ileus (7)  Ileal perforation (1)  Osteomyelitis (2)  Hepatitis (19)  Cholecystitis (3)  SIADH (7)  Bone marrow suppression (8)  Haemolysis (1) |
| **Walia, 2005**  India  2001-2003 (36)  All ages  Hospital  Retrospective | - To understand the current disease epidemiology, clinical presentation and antibiotic resistance patterns and analyze their impact on treatment and future preventive public health strategies.  - Patients selected from microbiology laboratory records.   - Patients with blood culture-confirmed typhoid fever included.   -Excluded from analysis: outpatients with culture-confirmed typhoid fever; patients who had previously received quinolones or cephalosporin or macrolides or chloramphenicol (17/110); patients who chose not to complete therapy at the hospital (3/110); and those who had chronic illness (2/110).  -Some complications defined.  -Complications reported from a subset of culture-confirmed typhoid fever cases included for analysis.  -Chloramphenicol, ampicillin, co-trimoxazole, cefixime, ceftriaxone, nalidixic acid, ciprofloxacin. | **Children**  N=68  **Adults**  N=20  Sn= n.r.  Age–Mean/Median= n.r.  DDA–77.2% > 1w  Pre-treatment=n.r. | **Children**  Cn=34 (50%)  Mn= 3 (4.4%)  **Adults**  Cn=7 (35%)  Mn= 1 (5%)  **All ages**  MCn= n.r.  AMR=n.r.  MDR=26 (29.5%)  NARS=63 (71.5%)  RFS=n.r. | **Children**  Encephalopathy (6)  Meningitis (4)  Pneumonia (4)  Pleural effusion (4)  Gastrointestinal bleeding (2)  Intestinal perforation & Peritonitis (2)  Hepatitis (17)  Jaundice (7)  Ascites (4)  Hepatic failure (1)  Hypotension (9)  Other (4)  **Adults**  Encephalopathy (2)  Pneumonia (2)  Gastrointestinal bleeding (1)  Intestinal perforation & Peritonitis (1)  Hepatitis (3)  Ascites (2)  Hypotension (2)  Acute renal failure (1)  Other (1) |
| **Kadhiravan, 2005**  India  2001-2003 (24)  All ages  Hospital  Prospective | - To evaluate the impact of infection with NARST on clinical outcomes in patients with typhoid fever.  -All consecutive patients including children with blood culture-proven typhoid fever, admitted to the hospital during study period and those treated as outpatients available for at least one follow-up visit, were prospectively recruited.  - Culture confirmed typhoid fever cases included in the analysis.  - Excluded: outpatients not available for follow-up visits.  - Some complications defined.  - Complications reported from all cases.  - Chloramphenicol, amoxicilin, co-trimoxazole, ceftriaxone, nalidixic acid, ciprofloxacin. | N=60  Sn= 40 (66.7%)  Age–Mean (SD)= 15 (9)  DDA–Median (IQR)=8 (4.8-14)  Pre-treatment= 26 (43%) | Cn= 11 (18.3%)  MCn=n.r.  Mn= 0  AMR= 47 (78.3%)  MDR= 22 (36.6%)  NARS= 47 (78%)  RFS= 0 | Gastrointestinal bleeding (1)  Encephalopathy (4)  Meningitis (1)  Hepatitis (4)  Myocarditis (1) |
| **Abucejo, 2001**  Philippines  1994-1997 (3)  All ages  Hospital  Retrospective | - To report some clinical and epidemiological observations on patients from whom *Salmonella* Typhi was isolated by blood cultures. - Review of laboratory records to identify culture confirmed typhoid fever cases. - Patients with culture confirmed typhoid fever. - Exclusion criteria not reported. - Complications not defined. - Total complications reported from all cases but not all listed by the authors. - Chloramphenicol, co-trimoxazole, ampicillin, ceftriaxone, ciprofloxacin, ofloxacin. | N=422  Sn= 255 (60.4%)  Age–Mean/Median= n.r.  DDF–57% > 1 week  Pre-treatment=71 (17%) | Cn= 77 (18.2%)  MCn=n.r.  Mn= 9 (2%)  AMR= 0  MDR= 0  NARS= n.t.  RFS=0 | Ileitis (21)  Psychosis (16)  Gastrointestinal bleeding (11)  Intestinal perforation (1) |
| **Kabra, 2000**  India  n.r.  Children  Hospital  Prospective | -To document clinical picture, complications, and the sensitivity pattern of the isolates at Ahmedabad, India.  -Consecutive children with typhoid fever diagnosis admitted to the hospital.  -Patients with culture confirmed typhoid fever.  -Exclusion criteria not reported.  - Some complications defined.  -Complications reported from all cases.  -Chloramphenicol, co-trimoxazole, ampi/amoxy, cephalexin, gentamicin, furazolidone, ceftriaxone/cefotaxine, ciprofloxacin | N=100  Sn= 52 (52%)  Age– 26% < 5y;  60% 5-10y;  14%>10y  DDA–62% >1w  Pre-treatment= n.r. | Cn= 40 (40 %)  MCn=n.r.  Mn= 0  AMR= 80 (80%)  MDR= 80 (80%)  NARS= n.t  RFS= 0 | Encephalopathy (18)  Gastrointestinal bleeding (14)  Hepatitis (4)  Acalculous cholecystitis (4)  Bowel perforation (3)  Nephritis (2) |
| **van den Bergh, 1999**  Indonesia  1989-1990 (n.r.)  Adults  Hospital  Prospective | - To see what have been achieved by the introduction of antibiotics treatment in the management of typhoid fever in Indonesia. - Patients recruited during an observational study. - Patients with blood/marrow culture confirmed typhoid fever. - Excluded: typhoid fever patients with incomplete medical data (1/106). - Some complications defined. - Complications reported from all cases.   -Antimicrobial susceptibility analysis not reported. | N=105  Sn=50 (47.6%)  Age–Mean (range)= 22(14-60)  DDA=n.r.  Pre-treatment=n.r. | Cn=14 (13.3%)  MCn=0  Mn=5 (5%)  AMR=n.r.  MDR=n.r.  NARS=n.r.  RFS=n.r. | Stupor or coma (3)  Bleeding or perforation (3)  Sepsis syndrome±shock (10)  Pneumonia (10)  Myocarditis (6) |
| **Khan, 1999**  South Africa  1993-1995 (37)  All ages  Hospital  Retrospective | - To examine the influence of sex in determining the clinical features, laboratory findings, and complications of typhoid fever. - Review of medical files of culture confirmed typhoid fever cases. - Patients with blood culture confirmed typhoid fever. - Excluded: medical records missing (9).   - Some complications defined.  - Total complications reported from all cases but not all listed by the authors.  -Antimicrobial susceptibility analysis not reported. | N=102  Sn= 46 (45.1%)  Age-Mean (SD)  Males, 17.9 (12)  Females, 22.5 (5.9)  DDA-Mean (SD)  Males, 10.3 (7.3)  Females, 6.7 (5.9)  Pre-treatment=n.r. | Cn=39 (38%)  MCn= n.r.  Mn=1 (1%)  AMR=n.r.  MDR=n.r.  NARS=n.r.  FRS=n.r. | Intestinal perforation (5)  Glomerulonephritis (12)  Hepatitis (9) |
| **Khosla, 1998**  India  1991-1992 (24)  Adults  Hospital  Prospective | - To study drug resistance in typhoid fever cases and their morbidity and mortality. - All patients suspected of typhoid fever. - Patients with culture confirmed typhoid fever. - Exclusion criteria not reported. - Complications not defined. - Complications reported from all cases. - Chloramphenicol, co-trimoxazole, ampicillin, tetracycline, amoxycillin, gentamicin, ciprofloxacin. | N=180  Sn= 125 (69.4%)  Age–Mean/Median=n.r.  DDA=n.r.  Pre-treatment=n.r. | Cn=51 (28.3%)  MCn=n.r.  Mn=12 (6.7%)  AMR= 124 (68.6%)  MDR= 18 (10%)  NARS=n.t.  RFS=5 (3%) | Peripheral cirulatory failure (2)  Myocarditis (10)  Neuropsychiatric complications (24)  Hepatitis (2)  Gut perforation (5)  Paralytic ileus (5)  Haemorrhage (4) |
| **Secmeer, 1995**  Turkey  1982-1992 (120)  Children  Hospital  Retrospective | - Present patients with ST infections, including signs and symptoms, clinical picture, laboratory findings, complications and the incidence of resistance to antimicrobial agents. - Review of medical files of culture positive typhoid fever patients. - Patients with culture confirmed typhoid fever. - Exclusion criteria not reported. - Complications not defined. - Total patients with complications not reported and only few listed by the authors. - Ampicillin, chloramphenicol, trimethoprim-sulfamethoxazole, cefoperazone, ceftriaxone. | N=27  Sn=16 (59.3%)  Age–Mean (range)   - 1. (15 days-13 years)   DDA=n.r.  Pre-treatment=n.r. | Cn=n.r.  MCn=n.r.  Mn=4 (14.8%)  AMR= 3 (11.1%)  MDR=n.r.  NARS=n.t.  RFS=n.t. | Subdural effusion (1)  Hemolytic anemia (1)  Encephalopathy (3) |
| **Rajajee, 1995**  India  1991–1992 (21)  Infants <3  Hospital  n.r. | - To present our experience with the patterns of clinical presentation and complications in MDRST in infants with blood culture positive S. Typhoid infection. - Recruitment/selection procedures not described. - Culture confirmed typhoid fever cases. - Exclusion criteria not reported. - Complications not defined. - Total patients with complications reported. - Chloramphenicol, ampicillin, co-trimoxazole, ceftriaxone, ciprofloxacin, amikacin, kanamycin, gentamycin, netromycin, ceftazidime, cephaloridine, carbenicillin, cefuroxime, cefotaxime. | N=71  Sn=n.r.  Age <3  DDA=n.r.  Pre-treatment=n.r. | Cn=33 (46%)  MCn=n.r.  Mn= 2 (2.8%)  AMR= 43 (60.5%)  MDR=n.r.  NARS=n.t.  RFS=0 | Meningitis (3)  Marrow hypoplasia (6)  Liver abscess (1)  Nephritis (2)  Pneumonia (1)  Hypothermia (10)  Cholecystitis (7)  Cyanotic episodes (3) |
| **Rasaily, 1994**  India  1990-1992, (24)  Children  Hospital  Prospective | -To study the epidemiological, bacteriological and clinical profiles of typhoid fever in children.  -Children of both sexes up to the age of 12 years suffering from clinically suspected typhoid fever (sustained temperature > 5 days but no signs and symptoms suggesting other infections), irrespective of severity and admitted to the hospital during the first five days of the week during the study period were enrolled in the study.  - Only MDR culture confirmed typhoid fever cases included for analysis.  - Excluded: MDR typhoid fever cases with complications identified at hospital admission; culture confirmed cases fully sensitive to antimicrobials.  - Some complications defined.  - Complications reported from a subset (172/204 MDR cases) of MDR cases (204/221 culture confirmed typhoid fever cases).  -Amikacin, ampicillin, chloramphenico, ciprofloxacin, furazolidone, gentamicina, nalidic acid, norfloxacin, tetracycline, trimethoprim-sulphamethoxazole. | N=172  Sn= 100 (58.1%)  Age–Mean (SD), 6 (2.5)  DDA–Mean (SD), 16.4 (10)  Pre-treatment= n.r. | Cn= 11 (6.4%)  MCn=n.r.  Mn= 3 (1.7%)  AMR= 172 (100%  MDR= 172 (100%)  NARS= 4 (2.5%)  RFS= 0 | Gastrointestinal bleeding (1)  Abdominal distension (4)  Delirium and abnormal state of consciousness (4)  Tremor (1)  Difficulty of speaking (1) |
| **Olle-Goig, 1993**  Haiti  1988-1991 (43)  Adults ≥ 14  Hospital  Retrospective | - To describe the clinical presentation and evolution of Typhoid fever among adult patients diagnosed at the Albert Schweitzer Hospital. - Review of laboratory records to identify culture confirmed typhoid fever cases. - Patients with culture confirmed typhoid fever. - Excluded: patients with HIV, incomplete medical files. - Complications not defined. - Total patients with complications not reported and only few listed by the authors. - Susceptibility antimicrobial analysis not reported. | N=217  Sn= 110 (50.7%)  Age–Mean (SD)= 21.1 (6.7)  DDA–95% < 15 days of illness  Pre-treatment= n.r. | Cn=n.r.  MCn=n.r.  Mn=20/129 (9.25)  AMR=n.r  MDR=n.r.  NARS=n.r.  RFS=n.r. | Pneumonia (6)  Melena/red blood in stool (6)  Ileal perforation (4)  Psychosis (2)  Acute respiratory distress syndrome (1)  Renal failure (1)  Abortion (1)  Premature delivery (1) |
| **Rao, 1993**  India  1990-1991 (18)  n.r.  Hospital  n.r. | - To report the incidence of MDR *Salmonella* Typhi infection along with clinical response to therapy in a rural hospital in India. - Patients suspected with typhoid fever infection. - Culture confirmed typhoid fever patients. - Exclusion criteria not reported. - Complications not defined. - Total patients with complications not reported and only few listed by the authors. - Chloramphenicol, Ampicillin, Tetracycline, Gentamycin, Kanamycin, Amoxicillin, trimethoprim-sulfamethoxazole, Ciprofloxacin, Norfloxacin. | N=102  Sn=n.r.  Age=n.r.  DDA=n.r.  Pre-treatment=n.r. | Cn= n.r.  MCn=n.r.  Mn= 1 (1%)  AMR= 80 (78.4%)  MDR=80 (78.4)  NARS=n.t.  RFS= 0 | Intestinal perforation (1)  Hemorrhage (1)  Toxic megacolon (1)  Hepatitis (1)  Acute renal failure (1) |
| **Sharma, 1992**  India  n.r.  Children  Hospital  n.r. | -To highlight the clinical profile of multidrug resistant enteric fever and use of ciprofloxacin in its management.  -Recruitment/Selection not reported.  -typhoid fever cases included in the study described as blood culture-proven cases.  -Exclusion criteria not reported  -Complications not defined.  -Total patients with complications not reported and only few listed by the authors.  -Chloramphenicol, cotrimoxazole, ampicilin, gentamicin, norfloxacin. | N=65  Sn= 100 (58.1%)  Age–Mean (SD)  MDR group, 9.8 (3.3)  Non-MDR group, 6.8 (3.5)  DDA-Mean (SD)  MDR group, 15.8 (8.2)  Non-MDR group, 13.1(9)  Pre-treatment= n.r. | Cn= n.r.  MCn= 49 (75.4 %)  Mn= 2 (3.1%)  AMR= 42 (64.6%)  MDR= 42 (64.6%)  NARS= n.t.  RFS= 0 | Bleeding (2)  Encephalopathy (24)  Myocarditis (6)  Shock (17) |
| **Mishra, 1991**  India  1990 (10)  Children  Hospital  Prospective | - To present the features of an outbreak of MDR S. Typhoid. - Patients with a clinical diagnosis of typhoid fever. - Culture confirmed typhoid fever patients. - Exclusion criteria not reported. - Complications not defined. - Total patients with complications reported. - Antimicrobials used to assess multidrug resistant typhoid fever not defined. | N=50  Sn=n.r.  Age–Mean/Median n.r.  DDA=n.r.  Pre-treatment=n.r. | Cn=14 (28.2%)  MCn=n.r.  Mn=1 (2%)  AMR= n.r.  MDR=39 (78%)  NARS=n.r.  RFS=n.r. | Encephalopathy (6)  Hepatitis (3)  Bronchopneumonia (3)  Myocarditis (1)  Pleural effusion (1) |
| **Mukherjee, 1991**  India  1989-1990 (6)  All ages  Hospital  Prospective | - To study the differences, if any, in the clinical pattern, presentation, therapeutic choices and outcome of chloramphenicol resistant and sensitive typhoid fever. - Patients admitted to the hospital with suspected typhoid fever. - Patients with blood culture confirmed typhoid fever. - Exclusion criteria not reported. - Complications not defined.   -Total patients with complications not reported and only few listed by the authors.   - Chloramphenicol, ampicillin, cloxacillin, co-trimoxazole, cipro/norfloxacin, gentamicin, furazolidone. | N=46  Sn=32 (69.6%)  Age–Mean (range)  19 (3m-35y)  DDA– 76% > 1 week  Pre-treatment=n.r. | Cn=n.r.  MCn=n.r.  Mn= 6 (13%)  AMR= 32 (69.5%)  MDR= 31 (67.3%)  NARS=n.t.  RFS=0 | Pneumonia (3)  Myocarditis (2)  Intestinal perforation (1)  Intestinal haemorrhage (8)  Bone marrow suppression (5) |

1 Patients with complications reported

2 Patients with more than one complication reported

3 AMR= *Salmonella* Typhi strains resistant to at least one of the antimicrobials tested by the authors.

4 MDR=Multidrug resistant *Salmonella* Typhi strains.

5 NARS=Nalidixic acid resistant *Salmonella* Typhi strains.

6 FRS=Fluoroquinolone reduced susceptibility / resistant *Salmonella* Typhi strains.

n.t. not tested

n.r. not reported
